# Insights into Urological Cancer

**DOI:** 10.3390/cancers13020204

**Published:** 2021-01-08

**Authors:** Claudia Manini, José I. López

**Affiliations:** 1 Department of Pathology, San Giovanni Bosco Hospital, 10154 Turin, Italy; 2 Department of Pathology, Cruces University Hospital, Biocruces-Bizkaia Health Research Institute, 48903 Barakaldo, Spain

The year the Covid-19 pandemic appeared has been quite prolific in urological cancer research, and the collection of articles, perspectives, and reviews on renal, prostate, and urinary tract tumors merged in this *Urological Cancer 2020* issue is just a representative sample of this assertion. Urological malignancies nowadays remain a hot topic of translational oncologic research. These are quite common neoplasms in clinical practice, with a high impact on the economy. All of them rank in the top-ten list of human cancers and account for up to 33% of malignancies affecting the male population [1]. In addition, kidney, prostate, and bladder tumors display different pathogenetic mechanisms, and their varied diagnostic and therapeutic approaches represent a challenge for multidisciplinary clinical teams.

Finding useful biomarkers to manage these diseases represents a key point in modern oncology. A relevant advance in the field, i.e., the importance and limits of analyzing the extracellular vesicles as potential biomarkers in urological neoplasms, has been reviewed this year, illustrating the promising therapeutic expectancies of this approach in the near future [2]. Since most biomarkers currently focused on in the research in the bladder, kidney, and prostate cancer (DNA, microRNA, proteins, etc.) travel packaged within exosomes and ectosomes in the bloodstream, these cellular structures remain as promising potential targets for testing both in urine [3] and liquid biopsies [4].

The European Association of Urology, in some instances, together with other international associations, such as the European Association of Nuclear Medicine, European Society for Radiotherapy and Oncology, European Society of Urogenital Radiology, and International Society of Geriatric Oncology, has recently updated the guidelines for the management of kidney [5], prostate [6,7], and bladder [8] tumors. On the other hand, pathological updates in tumor staging [9] and grading [10] of genitourinary tumors also appeared in 2020.

The pathogenesis, histological spectrum, molecular alterations, prognosis, and therapy of renal cancer is a maze for urologists, oncologists, pathologists, and basic researchers [11]. Under the term “renal cancer”, several different diseases coexist, each one of them being heterogeneous by itself. In this complex context, an exhaustive and systematic update of renal cancer classification based on morphological, immunohistochemical, and molecular data was performed during 2020 by a panel of international experts belonging to the Genitourinary Pathology Society (GUPS) [12,13]. While the most efficient treatment for the non-clear cell renal cell carcinomas group remains poorly defined, recent trials advise for the use of nivolumab/cabozantinib alone or in combination with immune checkpoint inhibition as the elective strategy for advanced clear cell renal cell carcinomas (CCRCC) [5]. 

CCRCC is a paradigm of unpredictable intratumor heterogeneity, and this fact makes it especially difficult to find a successful therapeutic response for every patient [5]. The decision to use immune checkpoint inhibitors depends on the immunohistochemical evaluation of the PD-1/PD-L1 axis status in the intratumor inflammatory infiltrates, a subject that is controversial since insufficient or partial tumor analysis may provide inconclusive results [14]. A recent study, however, has obtained much more sensitive results in its evaluation using a new methodology called Förster Resonance Energy Transfer (FRET), which is based on the physical properties of the intervening molecules [15].

This *Urological Cancer 2020* Special Issue includes two articles and one perspective on renal neoplasia [16,17,18]. Schnetz et al. [16] analyzed the role of macrophage-secreted iron in tumor progression of patients with CCRCC, papillary renal cell carcinomas (PRCC), and chromophobe renal cell carcinoma (ChRCC). They have found that genes regulating iron homeostasis are associated with tumor stage and grade through the pro-tumorigenic activity of a specialized subset of macrophages present in the local microenvironment. The iron chelator EC1 seems to reverse the pro-tumorigenic effect of these macrophages scavenging iron in the local extracellular matrix. Mihalopoulos et al. [17] hypothesized that the quinazoline-based α1-adrenoreceptor-antagonists may have a direct therapeutic action in renal cancer and reviewed its mechanism of action in human disease, their antitumor effects in several neoplasms, and its potential therapeutic usefulness in renal cancer. Finally, Tetar et al. [18] described their results using the stereotactic magnetic-resonance-guided radiotherapy (MRgRT) in 36 patients with large renal tumors and concluded that this technique shows low toxicity and high local control of the disease.

Two large groups of patients with urothelial carcinomas are distinguished in the clinical practice, non-muscle invasive and muscle invasive, each one of them displaying a specific clinical approach and management. Apart from the classical prognostic parameters, such as tumor Stage and Grade [19], still valid, pathologists distinguish basal and luminal phenotypes in muscle-invasive urothelial carcinomas based on immunohistochemical and molecular profiles, which correlate with different molecular tumorigenic pathways, clinical evolution, and prognosis [20]. Some patients with advanced urothelial carcinoma may benefit from immune checkpoint blockade [21]. 

Two articles [22,23] and two reviews [24,25] dealing with urothelial tumors have been included in this collection of urological neoplasia. Chien et al. [22] described in a study of 635 patients, how the overexpression of the microtubule-associated protein 1b (*MAP1B*) is an independent prognostic factor with adverse clinical outcomes and shorter survival in both upper urinary tract and bladder urothelial carcinomas. The authors concluded that MAP1B could be used as an additional biomarker and then potentially targeted in the future. Kubon et al. [23] analyzed the mRNA of three immune markers (*CXCL9*, *PD-1*, and *PD-L1*) in a series of non-muscle invasive bladder urothelial carcinomas and demonstrated that increased levels of *CXCL9* mRNA are associated with longer overall and disease-free survivals. In addition, they have confirmed the survival benefit of high levels of *PD-L1* mRNA. 

The two reviews come directly from the clinical perspective. A multi-institutional and international group of urologists achieved a consensus to define and predict the complexity of transurethral resection and dissection of bladder tumors, a crucial issue to optimize and adapt human and technical resources to every clinical setting [24]. Most urothelial carcinomas are composed of clearly recognizable, though more or less differentiated, transitional cells. Manini and López [25] reviewed the varied morphology that urothelial tumors may eventually display with their respective characteristic pictures. The importance of its correct recognition relies on that some of them carry prognostic implications per se, a point that every pathologist should know. 

Prostate cancer usually presents as a localized (organ-confined) disease. Depending on several factors, radical surgery or radiotherapy are the two recommended treatments [26], but active surveillance [27] and focal therapy [28] have also been proposed in selected low-grade/low-volume cases. Another subset of patients presents with aggressive and disseminated disease at diagnosis [29]. In between these two extreme clinical settings, the third subset of patients is characterized by the development of only a few metastases (<5) along the clinical course of the disease, the so-called oligometastatic prostate adenocarcinoma. Oligometastatic prostate cancers are usually under-diagnosed because they do not present any specific clinical or histologic feature [30]. As a result, the on-time identification and application of any eventual treatment exclusively directed to the metastases remain a difficult challenge. 

*Urological Cancer 2020* contains three reviews [31,32,33] and seven articles [34,35,36,37,38,39,40] about prostate cancer. Sarkar et al. [31] reviewed the intimate mechanisms of angiogenesis in prostate cancer and their possible blockade to maximize benefits minimizing toxicity. Another interesting review revisited the cellular and molecular progression pathways of prostate cancer in different cell lines [32]. The prostate cancer associated with *PTEN*-deficiency deserves a special mention. It is well-known that *PTEN* loss is a key factor for cancer initiation in many organs, the prostate included. In this sense, a recent study showed that *PTEN* heterozygosity in *LKB1*-mutant mice promotes the development of a metastatic aggressive form of prostate cancer [41]. Bardis et al. [33] reviewed the applications of Artificial Intelligence to multiparametric magnetic resonance imaging in prostate cancer and its interaction with radiologists’ algorithms. 

Cattrini et al. [34] analyzed the epidemiological characteristics of more than 26,000 patients collected in 17 years to understand the effect on survival provided by the advances in therapy in de novo metastatic prostate cancer better. A dual-time point hybrid imaging [^68^Ga]Ga-PSMA-11 PET/CT has been implemented for staging and restaging 233 prostate cancer patients [35], showing a potential benefit to define improved algorithms with clinical applicability to detect the primary, recurrent, and metastatic cases better. Crumbaker et al. [36] performed a retrospective deep whole-genome sequencing in a series of 13 patients with prostate cancer, highlighting the extreme genomic complexity and heterogeneity. This information may be of help when making therapeutic decisions, for example, unveiling cases with alterations in PI3K, MAPK, and Wnt pathways or detecting losses in genes related to sensitivity to immunotherapy or with resistance to androgen therapy. As in many other cancers, the local microenvironment greatly influences prostate cancer cell evolution. In this sense, Karkampouna et al. [37] analyzed the importance of the stromal signatures found in xenograft models of metastatic prostate cancer, which correlated with clinical parameters, such as the Gleason score, metastasis progression, and progression-free survival.

DONSON (downstream neighbor of SON) mRNA expression was analyzed by Klümper et al. [38] in aggressive variants of prostate cancer. Upregulation of this gene related to cell cycle progression and genomic stability maintenance has been associated with clinical aggressiveness, metastases development, and androgen-deprivation resistance. The authors stressed that DONSON expression could be considered a robust prognostic biomarker in prostate cancer [38]. Androgen deprivation resistance is the final common step of many advanced prostate cancers. Li et al. [39] identified the key promoting role of KLF5, the transcription factor Krüppel-like factor 5, in the androgen-AR signaling of LNCaP and C4-2B prostate cell lines. The authors considered this effect as a potential target to develop new therapeutic strategies in castration-resistant prostate cancers. Transcriptomic and metabolomic analyses of LNCaP-C4-2 cell lines with depleted and increased CPT1A (carnitine palmitoyl transferase 1) expression, respectively, were investigated [40]. CPT1A plays an important role in the adaptation to stress and antioxidant production and is an enzyme involved in lipid catabolism and may be critically involved in promoting neuroendocrine differentiation in prostate cancer cells. The authors concluded that an excess of CPT1A is associated with prostate cancer progression and propose to target lipid catabolic pathways as an alternative therapeutic tool. 

To summarize, this *Urological Cancer 2020* collection contains a set of multidisciplinary contributions to the extraordinary heterogeneity of tumor mechanisms, diagnostic approaches, and therapies of the renal, urinary tract, and prostate cancers, with the intention of offering a representative snapshot of the current urological research.
